# Burden of influenza in Germany: a retrospective claims database analysis for the influenza season 2012/2013

**DOI:** 10.1007/s10198-015-0708-7

**Published:** 2015-07-05

**Authors:** Jennifer Haas, Sebastian Braun, Peter Wutzler

**Affiliations:** Xcenda GmbH, Lange Laube 31, 30159 Hannover, Germany; Institute of Virology and Antiviral Therapy, Jena University Hospital, Friedrich-Schiller University, Hans-Knöll-Str. 2, 07745 Jena, Germany

**Keywords:** Influenza, Claims data, Germany, Burden of disease, I10, I13

## Abstract

**Objective:**

Seasonal influenza occurs in annual epidemics. The virus can cause severe illness and concomitant diseases with the highest risk for children, the elderly, and individuals with disease dispositions. The study objective is to assess the influenza burden in Germany.

**Methods:**

This retrospective claims data analysis used the Health Risk Institute research database containing anonymized data of 4 million individuals. The study period comprised the influenza season 2012/2013 in which patients with documented influenza were identified. Disease frequency rates were calculated for a population with disease dispositions and a population not at high risk. Disease burden was assessed based on health-services utilization during the influenza season. Vaccine rates were calculated by identifying vaccinations.

**Results:**

We observed 65,826 patients with influenza, resulting in 1,160,646 documented influenza cases after extrapolation. Overall, otitis media and pneumonia was higher in the influenza-infected population compared to the non-influenza-infected population and especially high in children. Hospitalization cost amounted to €87,202,485 with a mean stay of 7 days, and total outpatient costs were €14,947,976. Vaccination rates were <4 % for children and 37 % for patients aged >60.

**Conclusions:**

Seasonal influenza can cause severe outcomes with hospitalizations and excess costs. Especially influenza-infected children are affected by concomitant diseases with higher disease burden. Furthermore, documented vaccination rates are quite low.

## Introduction

Seasonal influenza, an acute viral infection, usually occurs in the winter months and can easily be spread from individual to individual. In recent years, the seasonal epidemics have affected approximately 10–20 % of the world’s population [1]. According to the World Health Organization (WHO), yearly influenza epidemics lead to serious disease and death in high-risk populations [2]. In principle, influenza epidemics can affect every age group and cause mild-to-severe illness, whereas the highest risk of a severe course and concomitant diseases is associated with children, the elderly, and individuals with a disease disposition [3]. A 30–40 % rate of all influenza cases during an epidemic have been reported to be accounted for in children. Moreover, otitis media and pneumonia are the most frequent concomitant diseases in children [3–5]. Given the high annual morbidity, a substantial economic burden arises due to direct medical cost in the outpatient and inpatient sector. In addition, indirect cost caused by productivity losses due to incapacity to work pose a considerable economic burden [6].

Vaccinations can provide an effective tool for the containment of disease spread [7]. Currently, also against the background of an increased risk of infection for individuals with a disease disposition [4], the German Standing Committee on Vaccination (STIKO) recommends a yearly influenza vaccination for individuals aged 60 years or older and a vaccination for certain individuals with an underlying disease [8]. To date, there is no readily available data for influenza vaccination rates in Germany for the season 2012/13. Vaccination coverage for 2010/11 was estimated at 28.3 % in Germany [9].

The number of patients affected by an influenza infection and seeking health care may serve as an indicator for the impact of the seasonal burden. Due to the variety of individuals affected by an influenza epidemic, the severity of influenza and the vaccination frequency are important parameters [10]. In addition, the costs and productivity losses might be substantial [6].

To assess the impact of influenza on the health care system and estimate the burden of disease caused by influenza in Germany, a retrospective claims database analysis was performed. For this purpose, individuals with a documented influenza diagnosis were identified during the influenza season 2012/13. In terms of higher disease susceptibility, disease frequency rates were calculated for an at-risk population and individuals without specific risk profiles. Aside from the epidemiological analysis, health care resource use and cost were determined for pharmaceuticals, outpatient care, incapacity to work, and inpatient care. Furthermore, vaccination rates were approximated for the influenza season 2012/13.

## Methods

The study sample consisted of claims data from the Health Risk Institute (HRI), which includes claims of about 80 different health insurances (out of approximately 130 in Germany) and comprises the utilization of services on an anonymous patient by patient individual level. This research database comprises more than 4 million covered lives. The sample is representative for the German population in terms of age and gender.

According to the Robert Koch Institute (RKI), the influenza season 2012/13 covers the period of the 40th calendar week in 2012 until the 20th calendar week 2013 [11]. To identify all patients with a documented influenza during the influenza season 2012/13, the study period was from 01 October 2012 to 30 June 2013. This expanded time frame was chosen due to the practice of quarterly documentation of diagnosis in the outpatient setting. Patients were identified by the International Statistical Classification of Diseases, German Modification (ICD-10-GM) codes for influenza (J09, J10, J11). Patients were defined as influenza patients if they had an outpatient or inpatient diagnosis with at least one of the three influenza codes during the study period. In addition, the following quarter after the index quarter, defined as the quarter with the first documented ICD-10-GM code for influenza, was assessed for each patient individually. Except for patients diagnosed with influenza in the 2nd quarter of 2013 (01 April until 30 June) as per definition the influenza season 2012/2013 ended on 19 May 2013 and all patients had at least 6 weeks to recover from their influenza infection in the 2nd quarter of 2013. The identified patients were stratified by age groups.

To compare the frequency of influenza within an at-risk population and individuals without specific risk profiles the complete HRI research database was divided into two groups; an at-risk and a not-high-risk population. To assess the risk disposition, a 1-year pre-observation period before the beginning of the influenza season 2012/2013 was chosen. The pre-observation period included the 4th quarter 2011 until the 3rd quarter of 2012. Patients were defined as at-risk if they had at least one diagnosis of diseases of the respiratory system, the circulatory system, the genitourinary system, the nervous system (multiple sclerosis), or certain disorders involving the immune mechanism (including HIV). These diseases were derived from the recommendations of the German Standing Committee on Vaccination [12]. Furthermore, individuals were either required to be continuously insured for the pre-observation period and until the end of the influenza season or have been deceased during the influenza season. In addition, the identified patients were stratified by age groups.

The relative frequency of the most common concomitant diseases, otitis media and pneumonia, was evaluated and compared to individuals not infected with influenza. To calculate the relative frequency of otitis media, the ICD-10-GM codes acute otitis media were applied. The timeframe for the assessment of relative frequency was the influenza season including the 4th quarter of 2012 until the 2nd quarter of 2013. Otitis media was assessed in the group of individuals infected by influenza and also in individuals without an influenza infection in the season 2012/2013. Individuals assigned to the otitis media group without an influenza infection were not part of the influenza population.

Similar to the assessment of otitis media frequency, the relative frequency of pneumonia was evaluated by identifying individuals with an ICD-10-GM code for “bacterial pneumonia, not elsewhere classified” J15.* in the outpatient or inpatient sector during the 4th quarter of 2012 and 2nd quarter of 2013. Individuals were required to have at least one coding for bacterial pneumonia during this period and either be part of the influenza group or have no influenza infection during the influenza season 2012/2013. In accordance with the otitis media group, individuals assigned to the pneumonia group without an influenza infection were not part of the influenza population. Furthermore, the relative risk was calculated for both the otitis media and pneumonia group [13].

Healthcare resource utilization and costs were calculated from the perspective of the statutory health insurance for the influenza season 2012/2013. No co-payments or out-of-pocket payments were taken into account [14]. For each patient individually, the index and subsequent quarter of the influenza infection were assessed for resource use and cost. Direct costs were calculated separately for each domain—prescriptions, outpatient care, and inpatient care—based on the influenza-specific resource utilization. The medicinal treatment was identified by assessing the prescriptions based on ATC codes for analgesics (N02), antibiotics (J01), antitussives (R05D), M2-Membranprotein inhibitor (N04BB01), Neuraminidase inhibitor (J05AH02, J05AH01) and nose spray (R01A, R01B) [15]. Outpatient resource use included number of outpatient visits based on the documented uniform assessment standard (EBM) points during the observation period. Outpatient data is only available on a quarterly basis and each visit cannot be linked to a specific ICD-10-GM code. As a result, EBM codes were used to identify number of outpatient visits and costs during the observation period. Furthermore, the number of incapacity to work days due to influenza was assessed during the influenza season. The inpatient scope included the number of hospitalizations with an influenza coding as the principal or secondary diagnosis and the corresponding days. The amount of main and secondary influenza inpatient codes were all stratified by age groups. Total inpatient cost was extracted directly from the database and extrapolated. The incapacity to work days for influenza were assessed and an exemplary calculation of the productivity loss was performed; assuming an average cost per incapacity to work day of €176 based on the lost gross value [16].

To identify influenza vaccinations relevant for the season of interest, an observational period before and partly during the influenza season 2012/2013 was chosen, as most vaccinations for influenza take place during the winter months beginning in September [8]. The observation period comprised 01 July to 31 December 2012 and included all patients with at least one vaccination identified by codes for vaccination 89111 Influenza (standard vaccination) and 89112 Influenza (other indications).

The results were extrapolated to the German statutory health insurance (SHI) population. On 31, December 2012 the total German population comprised about 80,523,700 individuals according to the Federal Statistical Office [17]. The underlying claims data sample from the HRI research database is already adjusted for the age and gender distribution in the German population. Therefore, the extrapolation was calculated by multiplying the results with an extrapolation factor. This factor is determined through dividing the German population by the specific study sample given on 31, December 2012.

Confidence intervals with 95 % confidence level were calculated by applying the Clopper–Pearson interval. Furthermore, the results were adjusted for the German SHI population by calculating an adjustment factor of 0.8656 based on the ratio of SHI population 69,704,000 [18] to German population of 80,523,700.

## Results

### Influenza cases and rate calculations

Table 1 shows the rate of patients identified in the age groups ranging from 0 to 1 to above the age of 60. Rate in percent was calculated based on the underlying German SHI population. The highest rates of documented influenza are present in the age group of 2–6 years (3.2 %). Overall, 1.7 % of the SHI population had a documented influenza infection during the influenza season 2012/2013.

**Table 1:** Rate of patients with a documented influenza diagnosis and total patient counts stratified by age groups

Age groups	Rate in % (95 % CI)	Total^a^
0–1	2.47 (2.31; 2.64)	14,952
2–6	3.17 (3.09; 3.26)	91,951
7–12	2.43 (2.36; 2.50)	91,457
13–17	1.93 (1.87; 1.99)	67,778
18–34	1.93 (1.90; 1.96)	271,339
35–59	1.89 (1.87; 1.92)	496,218
≥60	0.68 (0.66; 0.70)	126,951
Total	1.67 (1.65; 1.68)	1,160,646

^a^The extrapolation is based on the sample size of 3,953,260 and an extrapolation factor of 17.63, adjusted to the SHI population with 86.56 %

**Fig. 1:**
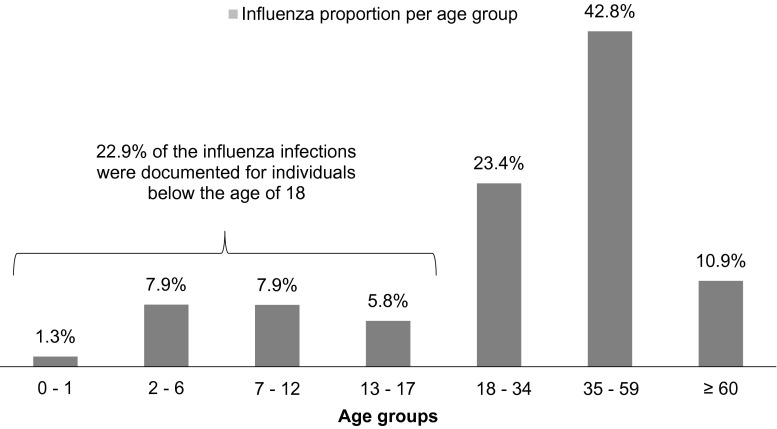
Influenza proportion per age group

In total, 65,826 documented influenza cases were observed in the HRI database and extrapolated to the SHI population. The extrapolation resulted in 1160,646 (95 % CI 1,330,667; 1,351,001) influenza patients during the 4th quarter 2012 until the 2nd quarter of 2013, adjusted for the SHI population. Analyses showed a similar rate of documented influenza in male and female patients for all age groups. Therefore, no further subgroups for gender are described. The following Fig. 1 comprises the distribution of influenza cases per age group based on the observed patient counts.

### At-risk population

**Fig. 2:**
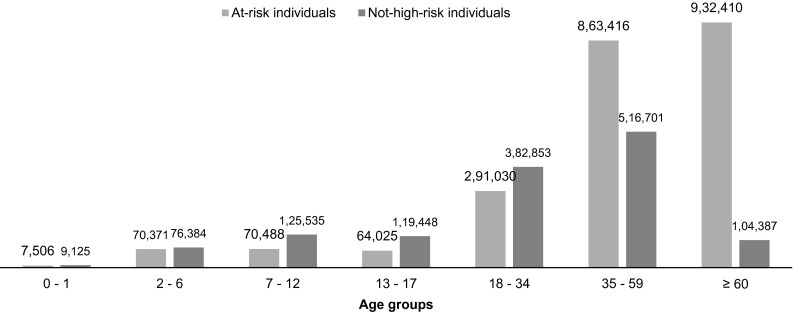
Distribution of at-risk and not-high-risk individuals in the HRI data pool

As described in the Methods section, the risk disposition was assessed during the 1-year pre-observation period before the beginning of the influenza season 2012/2013. In total, 3,633,679 individuals were available and continuously enrolled from the beginning of the pre-observation period until the end or deceased during the influenza season. The assessment according to ICD-10-GM coding resulted in 63 % at-risk individuals and 37 % not-high-risk individuals (Fig. 2).

To compare the influenza disease frequency in the at-risk and not-high-risk groups, the rate was calculated based on the underlying German SHI population, at-risk and not-high-risk, respectively. The following Table 2 comprises the calculated influenza frequency based on the SHI extrapolation and the corresponding patient counts with an influenza diagnosis. At-risk patients, especially children aged between 0 and 12 had a higher influenza disease rate than healthy children at the same age groups. Individuals above the age of 60 had the lowest disease rate in both groups, whereas children between 0 and 1 had the highest disease frequency with 4.0 and 2.8 % in both groups. Chi-square test was applied for significance testing—comparing the influenza disease frequency in the at-risk and the not-high-risk group—and resulted in significant *p* values for all age groups except for the overall influenza rate. Due to the distribution of individuals in the different age groups, the higher proportion of individuals in the age groups 35–59 and 60+ and the similar influenza rates within those groups, the overall influenza rate resulted in 1.70 % for the at-risk individuals with influenza and 1.72 % for the not-high-risk group, respectively. Therefore, the overall standardized influenza rate was calculated to adjust for the age group distribution. The overall age standardized influenza rate for the at-risk-individuals with influenza was 1.94 %, compared to the not-high-risk group of individuals with influenza with 1.41 %, reflecting the higher influenza rates in all age groups.

**Table 2:** Comparison of influenza frequency in the at-risk and not-high-risk group

Age groups	At-risk individuals		Not-high-risk individuals		*p* value*
	Rate of documented influenza in % (95 % CI)	Patient counts^a^	Rate of documented influenza in % (95 % CI)	Patient counts^a^	
0–1	4.04 (3.60; 4.5)	5,812	2.83 (2.49; 3.19)	4,949	<0.0001
2–6	3.98 (3.84; 4.13)	53,769	2.73 (2.62; 2.85)	40,034	<0.0001
7–12	3.18 (3.05; 3.31)	42,989	2.17 (2.09; 2.26)	52,331	<0.0001
13–17	2.51 (2.39; 2.64)	30,846	1.72 (1.64; 1.79)	39,305	<0.0001
18–34	2.41 (2.36; 2.47)	134,778	1.76 (1.72; 1.81)	129,503	<0.0001
35–59	2.13 (2.10; 2.16)	353,020	1.68 (1.64; 1.71)	166,276	<0.0001
≥60	0.71 (0.69; 0.73)	126,856	0.42 (0.38; 0.46)	8,364	<0.0001
Total	1.70 (1.68; 1.71)	748,070	1.72 (1.70; 1.74)	440,762	0.0673

^a^The extrapolation is based on a sample size of 3,633,679 and an extrapolation factor of 19.18, adjusted to the SHI population with 86.56 %

* Chi-square test

### Relative frequency of otitis media and pneumonia

**Fig. 3:**
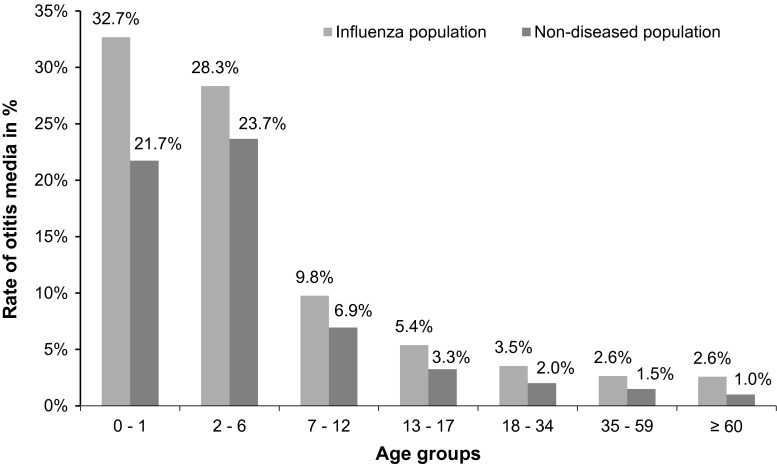
Otitis media frequency rate—comparison between influenza population and non-diseased population

As previously described the calculation of relative disease frequency for otitis media and pneumonia was performed. This resulted in an increased frequency of otitis media compared to the non-diseased population, especially for children aged between 0 and 6. The following graph shows that individuals with a documented influenza diagnosis had a higher rate of otitis media disease than individuals without an influenza diagnosis during the observation period. Furthermore, a relative risk of 2.04 [95 % CI 1.9809–2.1068; *p* < 0.0001] was calculated for the association with influenza (Fig. 3).

Similarly, the assessment of pneumonia frequency resulted in a higher number of pneumonia cases, defined as either pneumonia due to influenza or bacterial pneumonia, during the influenza season. A relative frequency of 2.5 % for the influenza population and 0.2 % for the population not affected by influenza was identified. Children between the age of 0 and 1 had a relative frequency of 4.5 % in the influenza population and 0.2 % in the non-diseased population. The relative frequency was highest for patients above the age of 60 (5.5 %). Individuals with influenza were more likely to contract pneumonia than individuals without an influenza diagnosis during the observation period. This is also the case when only comparing bacterial pneumonia occurrence between the two patient groups and excluding codes for influenza induced pneumonia. Individuals with an influenza infection had a higher rate of bacterial pneumonia in all age groups. The highest rates were observed in children aged 0–1 (0.8 vs. 0.2 %) and adults above the age of 60 (0.7 vs. 0.4 %) compared to the non-diseased population. The relative risk of acquiring a bacterial pneumonia was almost double [1.84 (95 % CI 1.5870–2.1273; *p* < 0.0001)] for influenza patients.

## Resource use and cost

### Inpatient care

Over 65 % of the inpatient visits were caused mainly by influenza, as influenza was the principal diagnosis. Especially, for the age groups 2–6, 7–12, and 13–17 the proportion of influenza principal diagnosis codes was over 80 %. The comparison of mean period per hospitalization with influenza as a principal diagnosis showed that children aged between 0 and 1 were in hospital for 5.2 days, in comparison to individuals aged 60 and above with an average of 10.4 days caused mainly by influenza.

Overall, 14,952 influenza-related hospitalizations were identified during the influenza season. These hospitalizations amounted to 150,436 days and total cost of €87,202,485 for the SHI population leading to cost per day of €579.66 for an influenza-related hospitalization. The average cost per inpatient case treated was €5,832.18.

Patients above the age of 60 were in hospital with a mean of 13.1 days while children aged between 2 and 6 were in hospital for 6.6 days on average. Overall, the mean duration of stay was 10.1 days for an influenza-related hospitalization.

### At-risk versus not-high-risk group

In context of the previously described risk analysis for influenza, the rate of inpatient visits was higher in all age groups for the at-risk population compared to the not-high-risk group. Children below the age of 18 and individuals above the age of 60 had the highest rate of influenza-related hospitalizations. For children below the age of 1, the rate of influenza-related hospitalizations was highest with 4 % for the at-risk individuals compared to 3.1 % for the not-high-risk individuals. The biggest difference between the calculated rates was in the group of individuals aged 60 and above (3.6 vs. 1.8 %).

### Outpatient care

Based on the individual observational period, influenza-related resource use and cost were assessed. In line with the determined ATC codes for pharmaceutical treatment, 41.92 % of the influenza patients had a prescription for antibiotics. Costs were highest for antibiotics and analgesics amounting to €10,124,474 and €6,243,561, respectively. In total, the influenza-related pharmaceutical cost was €19,846,172 for the influenza season 2012/2013 based on the SHI population in Germany. The following Table 3 comprises the calculated rate of pharmaceutical use based on the number of identified patients with a prescription. In addition, the corresponding cost of the influenza-related prescriptions were extrapolated.

**Table 3:** Resource use and cost of influenza-related pharmaceuticals

Substance	Rate in % (95 % CI)	Total cost in €^a^ (95 % CI)
Analgesics	18.75 (18.42; 19.08)	€6,243,561 (€6,223,951; €6,263,212)
Antibiotics	41.92 (41.43; 42.42)	€10,124,474 (€10,100,273; €10,148,712)
Antitussives	10.22 (9.97; 10.46)	€1,178,363 (€1,169,521; €1,187,254)
M2-Membranprotein inhibitor	0.03 (0.02; 0.05)	€10,168 (€9,355; €11,033)
Neuraminidase Inhibitor	3.55 (3.41; 3.70)	€1,160,684 (€1,151,907; €1,169,510)
	0.05 (0.03; 0.07)	€14,766 (€13,783; €15,801)
Others (nose spray)	12.51 (12.24; 12.78)	€1,114,015 (€1,105,414; €1,122,665)
	0.00 (0.00; 0.01)	€142 (€61; €279)
Total	€19,846,172 (€19,815,166; €19,877,200)	

^a^The extrapolation is based on a sample size of 3,953,260 and an extrapolation factor of 17.63, adjusted to the SHI population with 86.56 %

During the influenza season 2012/2013, 7,682,046 (95 % CI 7,660,540; 7,703,592) outpatient visits and total cost of €259,044,288 [95 % CI €258,926,618; €259,161,988] for the extrapolated SHI influenza population occurred. This amounted to an average cost per outpatient visit of €224 and 5.7 outpatient visits on average within the observation period. In addition, the resource use and cost of influenza antibody tests were assessed and extrapolated. EBM codes for Influenza tests amounted to 35,581 coded tests and costs of €542,661. Thorax examination resulted in a cost of €383,342.

The assessment of the resource use and cost resulted in total cost for the influenza season 2012/13 of €366,092,945 [95 % CI €365,867,736; €366,318,254] based on the SHI population. The highest cost were calculated for outpatient care (€259,044,288), followed by inpatient care (€87,202,485) and pharmaceuticals (€19,846,172).

### Exemplary calculation of the cost of productivity losses

Out of the influenza population with 65,826 individuals, 23,419 had incapacity to work caused by influenza. In total, after extrapolation, this resulted in 3,886,210 days of sick leave with a documented influenza diagnosis and a mean duration of incapacity to work of 8.15 days. The exemplarily calculated cost of productivity losses resulted in €683,972,960 for the influenza season 2012/2013 assuming cost per incapacity to work day of €176 [16].

### Vaccination rates

Based on documented vaccinations during the 3rd and 4th quarter of 2012, vaccination rates ranged between 1 and 37 % for the different age groups. Children aged 0–1 had the lowest vaccination rate with 1.3 %. Individuals above the age of 60, where an influenza vaccination is indicated, had the highest vaccination rate with 36.8 %. The extrapolation resulted in 9,500,740 vaccinated individuals in the SHI population with an overall vaccination rate of 13.6 %. The following Table 4 comprises the vaccination rates and patient counts with an influenza vaccination.

**Table 4:** Vaccination rates and patient counts

Age groups	Vaccination rates (95 % CI)	Extrapolation of individuals^a^
0–1	1.29 (1.18; 1.42)	7,812
2–6	2.80 (2.72; 2.89)	81,036
7–12	3.66 (3.58; 3.74)	137,354
13–17	2.82 (2.74; 2.89)	98,823
18–34	2.28 (2.24; 2.31)	319,729
35–59	7.51 (7.47; 7.55)	1,963,168
≥60	36.76 (36.65; 36.87)	6,892,817
Total	13.63 (13.60; 13.66)	9,500,740

^a^The extrapolation is based on a sample size of 3,961,887 and an extrapolation factor of 17.59, adjusted to the SHI population with 86.56 %

Over 70 % of the vaccinations were placed with individuals above the age of 60. In total, only 3.4 % of the vaccinations were administered to children under the age of 18.

## Discussion

The use of German claims data from a large research database covering approximately 4 million lives serves as a sophisticated approach to assess the burden of disease from a health economic perspective. To assess the influenza season 2012/2013, three study populations were identified covering patients diagnosed with influenza, individuals with a disease disposition and patients with an influenza vaccination. Overall, 1,160,646 influenza patients after extrapolation were identified in the SHI with total cost of €366,092,845 for this influenza season. When including the exemplarily calculated cost for productivity losses, the total cost was €1,050,065,805. In this section these results were compared and validated with the existing evidence for the influenza season 2012/2013 in Germany.

### Burden of disease

In Germany, direct and indirect medical costs of diseases caused by influenza viruses have been estimated to be 1–2.5 billion euros [19]. However, to date, no publications are available assessing the direct and indirect cost of the influenza season 2012/13.

The most recent data for the influenza season 2012/2013 is based on the epidemiological report from the German influenza sentinel system (AGI) of the Robert Koch Institute covering the time period of the 40th calendar week 2012 until the 20th calendar week 2013. The AGI registers acute respiratory tract infections in the winter seasons. In this system, the presence of influenza virus is monitored through syndromic sentinel surveillance by defining acute respiratory diseases (ARD) as acute pharyngitis, bronchitis or pneumonia with or without fever. Season 2012/13 included 834 physicians in 652 sentinel practices, which is 1 % of the primary care physicians in Germany. A sub-group of participating physicians swab patients with acute respiratory tract infections and send samples for testing to the national influenza reference center. Electronic data of patients was available from 108 sentinel practices. These tracked the ICD-10-GM codes J00 to J22, J44.0 and B34.9 [8, 11, 20].

Based on the Infection Protection Act (IfSG), a total of 66,000 laboratory-confirmed acute influenza disease cases and 10,700 laboratory-confirmed inpatient cases were reported. The sentinel estimations calculated 7.7 million excess outpatient consultations, 4.3 million excess cases of incapacity to work/bed rest, and 32,000 excess hospitalizations [11].

In comparison, we calculated 1,160,646 influenza patients with 7.7 million influenza-related outpatient visits, 3.9 million days of incapacity to work, and 14,952 hospitalizations during the influenza season.

An estimation of the AGI concerning the number of the overall influenza patients is missing and therefore a comparison with our 1,160,646 influenza patients is limited. Our study design is based on verified diagnosis codes and not on laboratory-confirmed disease. However, the RKI states that only a small number of patients with influenza-similar symptoms are laboratory screened and an underestimation based on the IfSG data is to be expected [11]. Yet the number of influenza-related outpatient visits is comparable between our study results and the estimation of the AGI. However, the days of incapacity to work are lower in our study population. This might be due to the fact that our database does not include sick leave for schoolchildren or bed rest. Furthermore, the number of hospitalizations in our study is almost half the amount as estimated by the AGI. The RKI states that the number of hospitalizations especially for infants might be overestimated as different co-circulating pathogens could be the cause of infection and therefore for hospitalization. This might be partially due to the fact that the AGI estimation is not based on inpatient claims but on information given by the physician if the patient was further transferred to hospital [11].

In general, the surveillance system is based on a less restrictive definition, as it refers to acute respiratory infection partly based on more ICD-10-GM codes for a possible influenza infection than our study design [21].

### At-risk population

The assessment of an at-risk and not-high-risk population during the influenza season 2012/13 was performed with a wide, conservative scope to identify any individual who might be at risk and profit from an influenza vaccination. The majority of individuals in the database (63.3 %) was thereby defined as being at risk. Out of these, 5.8 % were aged between 0 and 17 years. The lowest rate of being at-risk was identified for individuals below the age of 1 year, which could also be due to the shorter pre-observation period. In contrast, a recently published cohort study from the German Health Interview and Examination Survey for Children and Adolescents assessed chronic and vaccine-preventable diseases from 2009 to 2012. Vaccine-preventable diseases included chickenpox, pertussis, and measles. Chronic diseases were defined as migraine, cardiac disease, diabetes, epilepsy, febrile convulsion, and other [22]. This resulted in chronic diseases in 16.2 % of children aged 0–17 years. Children were divided into age groups from 0 to 2, 3 to 6, 7 to 10, 11 to 13, and 14 to 17. The highest prevalence rate for chronic diseases was within the 14–17 age group. The lowest prevalence rates were found for children aged 0–2 years with 10.1 % for boys and 7.7 % for girls, respectively [22]. However, this study is only remotely comparable to our results as the information is based on telephone interviews with the parents and we applied a broader approach to identify an at-risk population. Furthermore, the assessed age groups differ from our analysis.

### Relative frequency of otitis media and pneumonia

As previously described, the calculation of relative disease frequency for otitis media and pneumonia resulted in an increased prevalence for the diseased population in comparison to the non-diseased population and especially for children aged between 0 and 6 years. The otitis media frequency was over 30 % for infants aged 0–1 year. A relative frequency for pneumonia of 2.5 % for the influenza population and 0.2 % for the population not affected by influenza was identified. Children between the age of 0 and 1 had a relative frequency of 4.5 % in the influenza population and 0.2 % in the non-diseased population, suggesting a higher disease burden for children with influenza.

Heikkinen et al. assessed the highest average annual rate of influenza among children below the age of 3, of these 39.7 % developed acute otitis media as a complication [4]. Peltola et al. calculated prevalence rates of acute otitis media and pneumonia in Finland, showing that 24 % of the children developed acute otitis media, whereas 9 % of the individuals aged <17 developed pneumonia [23]. Similarly, Neuzil et al. identified the highest rates of acute otitis media and lower respiratory tract disease among children below the age of 2. In addition, acute otitis media was found in over 50 % of patients hospitalized with a confirmed influenza illness [24].

The comparison of published literature with our relative frequency rates of otitis media and pneumonia shows our results are in line with calculated prevalence rates and influenza can be associated with predisposing acute otitis media.

### Vaccination rates

In general, the calculation of vaccination coverage has been performed by using billing data and these results can be validated against available data from other studies [25]. The vaccination rates calculated in our study were highest for individuals aged 60 and above and lowest for infants aged 0–1 years. To date, there is no published literature on the influenza vaccination rate and cost in Germany for 2012. The cost for vaccination can be estimated by multiplying the cost per dosage with the number of individuals vaccinated. According to the prices given in the LAUER Taxe, individuals aged between 2 and 6 years receive “live attenuated influenza vaccine (LAIV)” at €23.49 per dosage and all others receive “trivalent inactive influenza vaccine (TIV)” at €6.27 per dosage [Lauer Taxe]. In total, estimated cost for influenza vaccination are €60,965,073. The estimated cost is relatively low in comparison to the calculated direct cost of €366,092,845 for the influenza season 2012/13.

Damm et al. estimated the yearly baseline coverage for individuals in different age groups, starting with children below the age of 2 up to adults aged 70 years and over in Germany. As a result, children below the age of 1 were assumed to have no baseline vaccination coverage, while vaccination coverage rates ranged between 11 and 23.6 % for children aged between 1 and 17 years. Coverage was estimated to grow with increasing age and was highest for the elderly population [26].

Contrary to Damm et al. who calculated the yearly coverage, Böhmer et al. estimated the seasonal influenza vaccination uptake. Their study based on telephone interviews from the German Health Update (GEDA10) with *n* = 22,050 and a smaller GEDA10-follow up (*n* = 2,493) which were both stated to be representative of the general population aged ≥18 years living in Germany. Seasons 2008/09, 2009/10 and 2010/11 were assessed. The seasonal influenza vaccine uptake varied between 26.6 % in 2009/10 (*n* = 13,040) and 29.8 % in 2008/09. Individuals not assigned to the health care worker group and/or chronically ill group, had a vaccination rate of 14.8 % in season 2010/11 (*n* = 2,492). Vaccination rates were lowest for individuals aged 18–29 years both for the target and the non-target group [9]. Similar vaccination ranges were observed in several other studies conducting telephone interviews [27–29].

The only two studies using billing data in this field of research were conducted by Reuss et al. and Schröder-Bernhardi et al., the latter was based on the IMS Vaccine Analyzer covering 37 pediatric practitioners and 145 general practitioners for the vaccination season July to March 2011/2012 and the following vaccination seasons 2012/2013 and 2013/2014. The analysis was performed for children and adolescents aged 0–18. The results were extrapolated to the German population and resulted in vaccination rates of 4.3 % in 2011/2012, 3.8 % in 2012/2013, and 4.2 % 2013/2014 [30].

Reuss et al. determined influenza vaccination coverage in the 2004/05, 2005/06, and 2006/07 seasons based on billing data of the German associations of statutory health insurance physicians. Vaccinations were identified by codes for vaccination. Vaccinations were usually administered from September to December. The vaccination rate was 49 % for individuals above the age of 60. In all three assessed seasons, vaccination rates in the population aged above 60 were increased in relation to the individuals’ age. In the group of 60- to 64-year-olds, the average rate was 38 %; for the 65- to 69-year-olds, 46 % were stated; for the70- to 74-year-olds, the rate was 51 %; and for those older than 75, a vaccination rate of 54 % was reported [31].

The additional literature search revealed that there are no readily available vaccination coverage rates obtainable for the season 2012/13 in Germany using a data source comparable to our study design. The most recent data comprises results based on the IMS Vaccine Analyzer referring to a different time frame (July to March 2012/2013) and might be biased due to selection of pediatricians and general practitioners taking part in an analyzer program not representable for the German population [30]. However, our calculated vaccination rates for children and adolescents aged 0–17 with 3.4 % are comparable to those published (3.8 % for 0–18 years old). In general consistent with our vaccination rates, the trend of higher vaccination coverage accompanied by higher age can also be observed in the literature. The previous passages clearly indicate that the comparability is limited due to the different influenza seasons, target populations and reported age groups [25, 29, 31, 32].

## Limitations

This study is limited by the nature of the data source. Claims data are recorded for accounting purposes and not for clinical research. As a result, it is not possible to characterize patients by clinical parameters such as disease severity or to see the physician’s intention for each intervention. Data on the incidence of influenza are limited by the difficulties inherent in distinguishing influenza from other influenza-like illnesses [33]. Furthermore, copayments and out-of-pocket payments are not included in the analyses which might underestimate the overall burden of disease.

## Conclusions

The results of our retrospective analysis suggest an increased disease burden of influenza for children aged between 0 and 17 years. In total, 22.9 % of the influenza infections were documented for individuals below the age of 18. In addition, the relative frequency of otitis media and pneumonia was highest for children aged 0–6 years. The relative risk of acquiring an otitis media infection with influenza was twice as high as for individuals without influenza. In comparison, the calculated vaccination rates were relatively low for all age groups and especially for children and adolescents under the age of 18 with 3.4 %. Moreover, the conservative approach of identifying at-risk individuals showed that these individuals had a higher influenza frequency than individuals without a disease disposition.

These results suggest that given the calculated low cost of influenza vaccination, a higher vaccination rate might lower the disease burden for children and individuals with a disease disposition and decrease the seasonal cost of influenza from a health insurance perspective.
